# Alkalization of Kraft Pulps from Pine and Eucalyptus and Its Effect on Enzymatic Saccharification and Viscosity Control of Cellulose

**DOI:** 10.3390/polym14153127

**Published:** 2022-07-31

**Authors:** Isabel Carrillo-Varela, Claudia Vidal, Sebastián Vidaurre, Carolina Parra, Ángela Machuca, Rodrigo Briones, Regis Teixeira Mendonça

**Affiliations:** 1 Centro de Investigación de Polímeros Avanzados, CIPA, Concepción 4030000, Chile; i.carrillo@cipachile.cl (I.C.-V.); r.briones@cipachile.cl (R.B.); 2 Centro de Biotecnología, Universidad de Concepción, Concepción 4030000, Chile; clauvidal@udec.cl (C.V.); svidaurre2017@udec.cl (S.V.); roparra@udec.cl (C.P.); 3 Facultad de Ciencias Forestales, Universidad de Concepción, Concepción 4030000, Chile; 4 Departamento de Ciencias y Tecnología Vegetal, Universidad de Concepción, Los Ángeles 4440000, Chile; angmachu@udec.cl

**Keywords:** cold caustic extraction, endoglucanase, Cellic CTec3, cellulose conversion, cellulose crystallinity, intrinsic viscosity

## Abstract

Bleached kraft pulps from eucalyptus and pine were subjected to cold caustic extraction (CCE) with NaOH (5, 10, 17.5, and 35%) for hemicelluloses removal and to increase cellulose accessibility. The effect of these changes was evaluated in enzymatic saccharification with the multicomponent Cellic CTec3 enzyme cocktail, and in viscosity reduction of pulps with the monocomponent *Trichoderma reesei* endoglucanase (EG). After CCE with 10% NaOH (CCE10) and 17.5% NaOH (CCE17.5), hemicellulose content lower than 1% was achieved in eucalyptus and pine pulps, respectively. At these concentrations, cellulose I started to be converted into cellulose II. NaOH concentrations higher than 17.5% decreased the intrinsic viscosity (from 730 to 420 mL/g in eucalyptus and from 510 to 410 mL/g in pine). Cellulose crystallinity was reduced from 60% to 44% in eucalyptus and from 71% to 44% in pine, as the NaOH concentration increased. Enzymatic multicomponent saccharification showed higher glucose yields in all CCE-treated eucalyptus samples (up to 93%) while only CCE17.5 and CCE35 pine pulps achieved 90% after 40 h of incubation. Untreated bleached pulps of both species presented saccharification yields lower than 70%. When monocomponent EG was used to treat the same pulps, depending on enzyme charge and incubation time, a wide range of intrinsic viscosity reduction was obtained (up to 74%). Results showed that eucalyptus pulps are more accessible and easier to hydrolyze by enzymes than pine pulps and that the conversion of cellulose I to cellulose II hydrate only has the effect of increasing saccharification of CCE pine samples. Viscosity reduction of CCE pulps and EG treated pulps were obtained in a wide range indicating that pulps presented characteristics suitable for cellulose derivatives production.

## 1. Introduction

Cellulose is the most abundant renewable polymer on Earth and is used as a raw material for production of several valuable products [1,2]. Each beta-D anhydroglucose unit of cellulose has three -OH groups that interact and form intra- and inter-molecular H-bonds. According to the H-bonds arrangement, crystalline and amorphous regions can be found. The proportion of these regions depends on the raw material and the treatments to which the cellulose has been subjected [3,4]. Likewise, the chains arrangement and the H-bond network within the crystalline regions can vary, giving rise to different polymorphs, cellulose I and II being the most studied forms [5]. Cellulose I (native cellulose) can be found as cellulose Iα and Iβ. Plant cellulose mainly consists of cellulose Iβ, whereas cellulose produced by primitive organisms crystallizes as Iα. Cellulose Iα has a one-chain triclinic unit cell and cellulose Iβ a two-chain monoclinic unit cell [6,7].

Cellulose I can be converted to cellulose II by regeneration and alkali treatments. During alkalization, fibers are converted into a swollen state, involving the formation of one or more soda-cellulose complexes [8,9,10,11]. When cellulose swells in an alkali solution, a complex with alkali ions and water molecules, known as Na-cellulose I is formed. When alkali is washed out of the Na-cellulose I complex, it is converted into Na-cellulose IV, which has an antiparallel chain packing with a two-chain monoclinic unit cell, where two water molecules are regularly located between the corner chains, forming hydrogen bonds with adjacent cellulose chains. Drying Na-cellulose IV (cellulose II hydrate) results in the stable cellulose II crystal [10,11]. During this conversion, the monoclinic crystal structure of cellulose I with two cellulose chains in a parallel orientation is converted to the crystalline structure of cellulose II with two antiparallel chains into the unit cell. It is known that cellulose II hydrate is less crystalline, more reactive, and more accessible to reagents than cellulose I [12,13,14].

Bleached kraft pulp has become an important source for producing cellulose-based products from wood such as biofuels, viscose, cellulose derivatives, and cellulose nanofibers, among others [6,7,15]. For these applications, the structure of cellulose has a complex but significant influence on the course of chemical or biological reactions [3]. In this context, enzyme-aided technology is a viable route to replace or improve traditional chemical and mechanical methods for cellulose transformation, contributing to the environmentally friendly development of cellulose-based products. The important advantages of the enzymatic treatments are related to their environmentally friendly nature, high substrate specificity, stereo- and regioselectivity, possibility of recycling, and activity in mild reaction conditions [16,17]. Cellulose saccharification can be affected by the presence of other compounds such as hemicelluloses and lignin, and due to the inherent crystalline structure of cellulose, factors that can inhibit or decrease the cellulase activity [17,18].

Dissolving-grade pulps, which are mainly used to produce cellulose-derivatives, refers to a source of high-purity cellulose, with a low amount or complete absence of hemicelluloses (<6%) and trace amounts of impurities [15]. Upgrading paper-grade pulp is a straightforward way to produce dissolving-grade pulp, where the removal of hemicelluloses, viscosity control, and increase in cellulose reactivity are fundamental [14,19]. Cold caustic extraction (CCE) has been reported as an effective treatment for hemicellulose removal [14,20]. However, depending on the alkali concentration, the supramolecular structure of cellulose and morphological features of fibers can be modified [9,21,22,23,24]. On the other hand, wood pulps can be obtained from different types of fibers, hardwoods such as eucalyptus and acacia, or softwoods, such as pine and spruce. Both differ in their chemical and morphological features. Lignin of hardwood consists mainly of different proportions of syringyl (S) and guayacil (G) units, while softwood lignin consists mainly of G units [25]. Xylans are the main hemicellulosic fraction of hardwoods, whereas mannans are common in softwoods [6]. Hardwoods have a more complex anatomy than softwoods. The basic tissue of hardwood xylem contains fibers, vessels, and radial parenchyma cells; while softwoods have only tracheid cells, which are larger than hardwood fibers, and have thinner cell walls and bigger lumens [6].

The aim of this work was to apply alkalization treatments to bleached kraft pulps of eucalyptus and pine wood, and evaluate how the changes occurred in pulp composition and cellulose structure affect the susceptibility of fibers to enzymatic hydrolysis for saccharification and in the decrease of the intrinsic viscosity for dissolving grade pulp purposes. For the first, a commercial enzymatic cocktail (Cellic^®^ CTec 3, Novozyme Inc., Curitiba, Brazil) was used and, for the later, commercial endoglucanases (EG) from *Trichoderma reesei* (Sigma-Aldrich, St. Louis, MO, USA). Hence, the results covered (1) a comparison of fiber biometry and the cellulose crystalline structure of eucalyptus and pine kraft pulps subjected to CCE treatments; (2) enzymatic saccharification of CCE-treated pulps; (3) EG effect on CCE-treated pulps for viscosity control; and (4) determination of relationships among fiber and cellulose features and the results observed during enzymatic treatments.

## 2. Materials and Methods

### 2.1. Materials

Sodium hydroxide (NaOH) pellets (CAS 1310-73-2), sulfuric acid (H_2_SO_4_) 95–97% (CAS 7664-93-9), copper(II) ethylenediamine (CED) solution (CAS 14552-35-3), HCl 37% (CAS 7647-01-0) and methylene blue (MB) stain (CAS 61-73-4) were purchased from Merck (Darmstadt, Germany). D-(+) glucose (CAS50-99-7), D-(+)-cellobiose (CAS 528-50-7) and D-(+)-xylose (CAS 58-86-6) analytical standards were purchased from Sigma-Aldrich (St. Louis, MO, USA). Commercial cellulase cocktail Cellic*^®^* CTec3 was obtained from Novozyme, (Curitiba, Brazil) and endoglucanase (EG) from *Trichoderma reesei* (CAS 9012-54-8) was purchased from Sigma-Aldrich (St. Louis, MO, USA).

### 2.2. Bleached Kraft Pulps

Bleached kraft pulps from *Eucalyptus nitens* and *Pinus radiata* were provided by a Chilean pulp mill company located in the Biobío Region (Chile). *E. nitens* pulp had cellulose content of 90%, xylans content of 9.8%, and intrinsic viscosity of 730 mL/g. *P. radiata* pulp had cellulose content of 92%, mannans content of 7.5%, and intrinsic viscosity of 510 mL/g.

### 2.3. Cold Caustic Extraction (CCE)

CCE of bleached pulps were carried out with NaOH solutions at 5, 10, 17.5, and 35% (*w/v*) with 10% pulp consistency in sealed polyethylene bags immersed in a thermostatic water bath at 30 °C for 1 h. The mixture was mechanically agitated by hand every 10 min. The pulp slurry was filtered using polyester fabric filter bag, thoroughly washed with distilled water, and centrifuged to 35% consistency. Alkali-treated pulps were coded CCE5, CCE10, CCE17.5, and CCE35, respectively. The conventional CCE process is mostly applied at 8–10% NaOH [26], since strong alkali concentration gives better cellulose swelling, which is favorable for removal of hemicelluloses [15]. In this work, the samples were subjected to CCE to study both the removal of hemicelluloses and to investigate the crystalline conversion of cellulose I to cellulose II. To ensure pulps with cellulose I, mixtures of cellulose I/II, and cellulose II were obtained, an experimental procedure used in a previous work was followed [24], where NaOH concentrations higher than 17.5% were needed to achieve complete conversion of cellulose I to II.

### 2.4. Chemical Characterization

Bleached and CCE treated pulps were characterized for their carbohydrates content according to the method previously published by Mendonça et al. [27], where 3 mL 72% H_2_SO_4_ was added to 300 mg pulp and hydrolysis was carried out at 30 °C for 1 h. Afterwards, the acid was diluted to 4% (*w/w*) with distilled water, and the mixture was transferred to a 250 mL Erlenmeyer flask and autoclaved for 1 h at 121 °C. The residual material was cooled and filtered through a porous glass filter (number 4). Hydrolysate was transferred to a 100 mL volumetric flask and volume made up to the meniscus. The concentrations of glucose, cellobiose, xylose, and mannose in the hydrolysates were determined by high performance liquid chromatography (HPLC, Hitachi-Merck, Tokyo, Japan) with an Aminex HPX-87H column (Bio-Rad, Hercules, CA, USA) operated at 45 °C and eluted at 0.6 mL/min with 5 mM H_2_SO_4_ through a refractive index detector. Analytical grade monomers of sugars were used as external calibration standards. The amount of sugar was converted to anhydro monomers using hydrolysis factors of 0.90 (glucose to glucans, and mannose to mannan), 0.92 (cellobiose to glucan) and 0.88 to xylose to xylans [28]. All analyses were carried out in triplicate.

### 2.5. Fiber Biometry

Average fiber length, fiber length proportion, fiber width, fines content, and kink index were determined using L&W Fiber Tester equipment (Lorentzen and Wettre, Stockholm, Sweden). During the analysis, the equipment was set to measure approximately 35,000 fibers of each sample, and fines were defined as elements with lengths of 0–0.2 mm to ensure they were not included in the final averaged fiber measurements [29].

### 2.6. Intrinsic Viscosity

The intrinsic viscosity of bleached kraft pulps and CCE-treated samples was measured by the capillary viscometer method according to the ISO 5352:2010 (E) standard, using copper (II) ethylenediamine solution (Merck CAS 14552-35-3). All measurements were carried out in triplicate.

### 2.7. Carboxyl Content

The carboxyl content was determined using the conductometric titration method [30], where 0.3 g pulp (dry weight) was dispersed in 55 mL of water and 5 mL of 0.01 M NaCl. The pH was adjusted to 2.5 by the addition of 0.1 M HCl, and the mixture was titrated with 0.01 M NaOH solution. NaOH solution was added at the rate of 0.1 mL/min until pH 11, and the conductivity was recorded at each point by a conductivity meter (HI 2315, HANNA Co, Seoul, Korea). The obtained titration curves showed two points of change, where A indicated the neutralization of the strong acid (HCl), and B represented the titration of the weak acid (carboxyl groups). The content of carboxyl groups was calculated via Equation (1):

(1) $$Carboxyl\;content\;\left(\frac{mmol}{g}\right)=\frac{\left[V\left(NaOH\right)\times C\;\left(NaOH\right)\right]}{g}\;$$

where V(NaOH) is the volume (mL) of NaOH solution used for titration between points A and B, C (NaOH) is the concentration of NaOH solution (M), and g is the dry mass of the sample used.

### 2.8. Specific Surface Area (SSA) from Methylene Blue (MB) Adsorption (SSA_MB_)

The specific surface area (SSA), defined as the total surface area of a solid material per unit of mass, is an important feature for sorption processes. e.g., an increased substrate SSA improves cellulose adsorption and make enzymatic process more effective [31,32]. Assuming that MB forms a monolayer of adsorbed molecules on the surface of sorbent particles, the SSA can be calculated using Equation (2):

(2) $$SSA_{MB}=\frac{q_{max}\;A_{MB}\;N_{a}\;}{MW_{MB}}$$

where $SSA_{MB}$ is the specific surface area (m^2^/g), $q_{max}$ is the maximum mass of adsorbed MB in the monolayer (g/g), $\;A_{MB}$ is the surface area occupied by one MB molecule (m^2^/molecule) (typically assumed to be 130 Å^2^ per molecule), $N_{a}$ is Avogrado’s number (6.02 × 10^23^ molecule/mol) and $MW_{MB}$ is the MB molar mass (355.89 g/mol) [33,34,35]. $q_{max}$ was estimated from the Langmuir isotherm model.

#### MB Adsorption and Langmuir Isotherm

The experimental data were obtained by performing a series of batch tests using 25 mg (dry weight) of each pulp sample in 50 mL MB solutions with different initial concentrations (60–300 mg/L) at pH 5.5. Experiments were performed in triplicate on an orbital shaker incubator at 25 °C, 100 rpm for 24 h. The MB concentration in solution was measured using a UV-Vis spectrophotometer (UV-1280, Shimadzu, Japan). The data were collected at 664 nm (the maximum absorbance peak observed for MB). The MB adsorption capacity of each sample was calculated using Equation (3):

(3) $$q_{e}=\frac{\left(C_{0}-C_{e}\right)\;V}{m}\;$$

where $C_{0}$ and $C_{e}$ are the initial and equilibrium concentrations of MB (mg/L), respectively, V is the volume of MB solution (L), and m is the oven dry mass of the pulp (g).

The nonlinear expression of the Langmuir isotherm model is given by Equation (4) [36]:

(4) $$q_{e}=q_{max}K_{L}\frac{C_{e}}{1+K_{L}C_{e}}\;$$

where $q_{e}$ and $q_{max}$ are the adsorption capacity at equilibrium and the maximum adsorption capacity (mg/g), respectively, $C_{e}$ is the concentration of MB solution at the equilibrium stage (mg/L), and $K_{L}$ is the Langmuir constant (L/mg), which relates to the free energy and affinity of adsorption. Langmuir parameters were estimated using the curve fitting functions of OriginPro 9.0 Software (OriginLab Corporation, Northampton, MA, USA).

### 2.9. X Ray Diffraction of Pulp Samples

Samples for X-ray diffraction (XRD) analyses were prepared by pressing 50 mg of freeze-dried samples in a hydraulic press to form pellets [37]. The pellets were placed in a sample holder, and X-ray diffractograms were collected after mounting the sample holder on a D4 Endeavor X-ray diffractometer (Bruker AXS Gmbh, Karlsruhe, Germany) with monochromatic Cu Kα radiation (λ = 0.154 nm) at 40 kV and 20 mA. The intensities were measured in the range of 5° < 2θ < 45°, with scan steps of 0.02°. Each sample was analyzed in duplicate. Curve fitting was performed using PeakFit software (Systat, Software Inc., San Jose, CA, USA) to identify individual peaks. The detailed deconvolution procedure can be found in Carrillo-Varela et al. [38]. In all cases, the F number was >45,000 (R^2^ < 0.997). The apparent XRD crystallinity index (CrI) of the samples was calculated from deconvoluted areas via Equation (5) [39]:

(5) $$CrI\;\left(\%\right)=\frac{A_{cryst}}{A_{total}}\times 100\;$$

where $A_{cryst}$ is the sum of crystalline band areas, and $A_{total}$ is the total area under the diffractogram.

From the sum of peak area of the same crystal system (ΣA_CI_ for cellulose I, and ΣA_CII_ for cellulose II), cellulose I and cellulose II percentages were calculated using Equations (6) and (7) [40,41]:

(6) $$\mathrm{Cellulose}\;I\;\left(\%\right)=\frac{\sum^{\text{​}}A_{\mathrm{CI}}}{\sum^{\text{​}}(A_{\mathrm{CI}}+A_{\mathrm{CII}})}\times \mathrm{CrI}$$

(7) $$\mathrm{Cellulose}\;\mathrm{II}\;\left(\%\right)=\frac{\sum^{\text{​}}A_{\mathrm{CII}}}{\sum (A_{\mathrm{CI}}+{\;A}_{\mathrm{CII}})}\times \mathrm{CrI}\;$$

The lateral crystallite size (*L*) was calculated from the Scherrer Equation (8) [42]:

(8) $$L=\frac{k\times \lambda }{\beta \times \cos\theta }\;$$

where *L* is the size of the crystallite (nm), *k* is the Scherrer constant (0.96), λ is the X-ray wavelength, β is the full-width half-maximum (FWHM) of the (200) reflection in radians, and θ is the Bragg angle corresponding to the (200) plane.

### 2.10. Enzymatic Multicomponent Saccharification of Pulps

Bleached and CCE-treated pulps were subjected to enzymatic hydrolysis using the commercial cellulase cocktail Cellic^®^ Ctec3 (Novozyme Inc., Curitiba, Brazil), which has endoglucanase and endoxylanase activity of 1562 and 2686 U/mL, respectively, determined by standard procedures with carboxymethyl cellulose (CMC) and birchwood xylan as substrates at pH 4, using the 3,5-dinitrosalicilic acid (DNS) to detect reducing sugar contents [43]. Saccharification was performed in 250-mL Erlenmeyer flasks with 2% pulp consistency in 0.05 M sodium citrate buffer (pH 4.8) at 50 °C for 90 h, in orbital shaker-incubator (Edmund Bühler, Hechingen, Germany), with an enzyme loading of 65.6 ECU (endocellulase units) and 112.8 EXU (endoxylanse units) per gram of pulp (dry weight basis). Samples were taken at different incubation times (up to 90 h). The concentration of glucose released in the medium was determined by HPLC following a similar procedure for analysis and quantification of the hydrolysate as described in Section 2.4. The hydrolysis yield (%) was calculated based on the amount of glucose released in the hydrolysate divided by the total amount of glucose in pulps assuming that most of the glucose comes from the cellulose fraction.

### 2.11. Endoglucanase Treatment of Pulps for Viscosity Reduction

Bleached and CCE-treated pulps were subjected to enzymatic treatment using commercial EG from *T. reesei* (Sigma-Aldrich, St. Louis, CA, USA). The EG activity was 10.81 U/mg using CMC and DNS method [43]. The treatment was performed in 250 mL Erlenmeyer flasks with 2% pulp consistency in 0.05 M sodium citrate buffer (pH 4.8) at 50 °C in an orbital shaker-incubator (Edmund Bühler, Hechingen, Germany). Two reaction conditions were performed: (1) with an enzyme loading of 5 ECU/g of pulp (dry weight) during 1 h, and (2) with an enzyme loading of 10 ECU/g of pulp (dry weight) during 2 h. At the end of each treatment, the enzyme was inactivated by water bath (90 °C) immersion of flasks for 10 min (procedure repeated 3 times for each sample). The pulp was washed with abundant distilled water, centrifuged to 35% consistency, and the intrinsic viscosity was determined.

### 2.12. Data Analysis

The data obtained was analyzed using OriginPro 9.0 Software (OriginLab Corporation, Northampton, MA, USA). Principal component analysis (PCA) was performed to reduce the dimensions of the dataset, retaining as much as possible of the data variation, and evaluate the spontaneous separation of samples. Correlation analysis among variables were performed using Pearson correlation coefficient (r). The statistical significance of Pearson index was also determined (*p*-value < 0.05).

## 3. Results and Discussion

### 3.1. Chemical Composition and Fiber Biometry

Chemical composition and fiber biometry of eucalyptus and pine pulps were reported in Table 1. Glucans content of eucalyptus and pine bleached pulps are similar (90–92%). Bleached eucalyptus pulp had a higher content of hemicelluloses than bleached pine pulp. However, after CCE10 treatments, pine pulp remains with a higher content of hemicellulose than eucalyptus. It could be attributed to a lower swelling capability of pine fibers that negatively affects the removal of hemicelluloses [26]. Regarding fiber biometry, eucalyptus fibers are shorter and thinner than pine. A higher number of fine elements were detected in eucalyptus, whose content increases with stronger alkali concentrations. Fines content in pine do not show the same decreasing trend, probably because they are already dissolved in the NaOH solution, and no new fiber fragments are produced after CCE treatments due to the large and wide structure of tracheids. As expected, weak points of fibers are also increased, which is demonstrated by the value of kink index (local deformations of fibers) [44].

**Figure 1:**
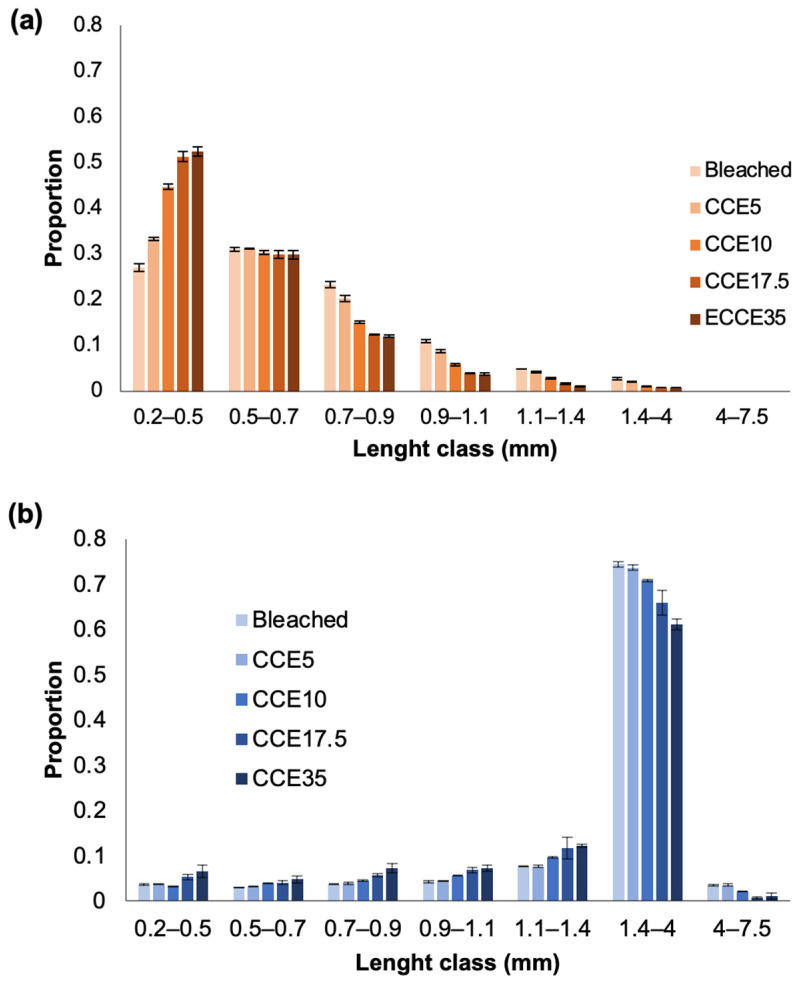
Fiber length classes distribution in (**a**) eucalyptus and (**b**) pine pulps after alkalization with different NaOH concentrations.

The proportion of fiber length (by size class) after each treatment with CCE is presented in Figure 1. In eucalyptus (Figure 1a), the bleached pulp contains 27% of the fibers between 0.2 and 0.5 mm of length, and 31% have a length between 0.5 and 0.7 mm. After each CCE treatment, the proportion of fibers ranging from 0.2 to 0.5 mm increased significantly, reaching 53% after CCE35. The proportion of fibers with 0.5–0.7 mm of length do not vary significantly. Additionally, the bleached pulp contained 23% of fibers with 0.7–0.9 mm of length, after CCE10 treatment, this fraction was less than 15%. In pine (Figure 1b) the bleached pulp contains the 75% of tracheid fibers with 1.4–4 mm of length, after each CCE, class length decreases to the 60%. As mentioned before, shorter fibers in pine are decreased probably because of dissolution, hence, the length proportion after each CCE treatment slightly increases from 0.2 to 1.4 mm classes.

### 3.2. Specific Surface Area by Methylene Blue (MB) Adsorption (SSA_MB_)

**Figure 2:**
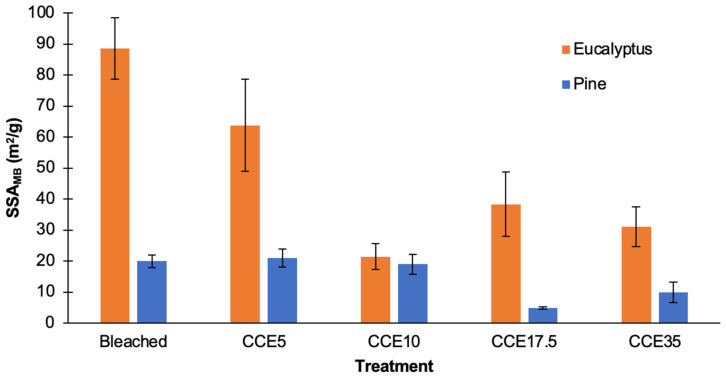
Specific surface area from MB adsorption (SSA_MB_) of bleached and CCE-treated kraft pulps from eucalyptus and pine.

The SSA of eucalyptus and pine pulps was determined using methylene blue (MB) adsorption method (Figure 2). Since cellulosic materials lose their inner porous structure when water is evaporated (hornification phenomenon) and conventional methods require sample drying [35], the SSA_MB_ was estimated in pulp wet state. Additionally, the experiments were conducted at pH 5.5 to reduce the effect of chemical adsorption of MB on the fiber surface [32]. SSA_MB_ results for eucalyptus samples ranged from 21 to 89 m^2^/g, while pine ranged from 5 to 20 m^2^/g, evidencing a meaningful difference on SSA_MB_ values in both species. The SSA_MB_ of bleached and CCE5 eucalyptus showed the highest values, that might indicate a significant influence of residual xylans to the negative charge of the surface fibers, adsorbing higher amounts of the cationic dye. Overall, eucalyptus samples have the highest total surface area of solid material per mass unit, which could facilitate the sorption of enzymatic and chemical reagents on the fiber surface [31].

### 3.3. Cellulose Crystallinity

**Figure 3:**
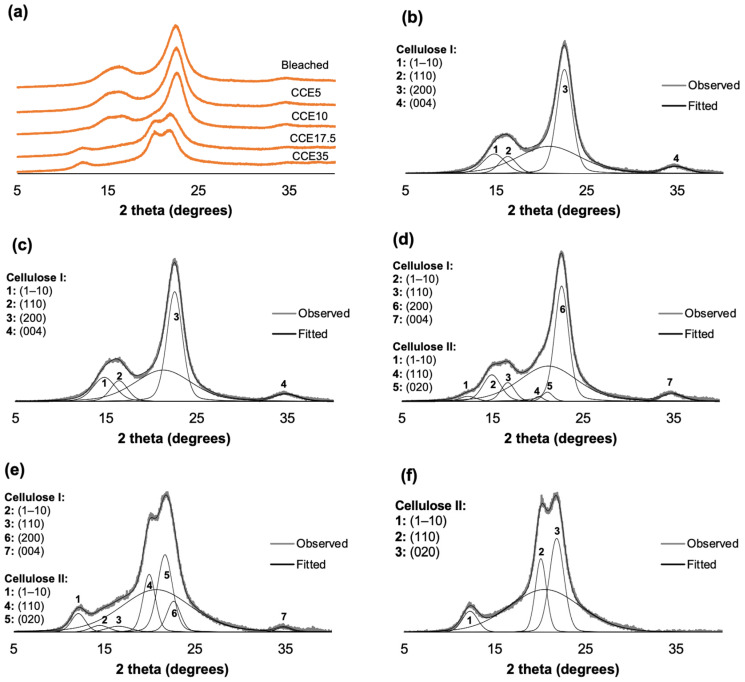
Eucalyptus XRD diffractograms. (**a**) XRD diffractograms of eucalyptus pulp after alkaline treatment, (**b**) curve-fitting of bleached kraft pulp, (**c**) curve-fitting of CCE5, (**d**) curve-fitting of CCE10, (**e**) curve-fitting of CCE17.5, and (**f**) curve-fitting of CCE35 eucalyptus sample.

**Figure 4:**
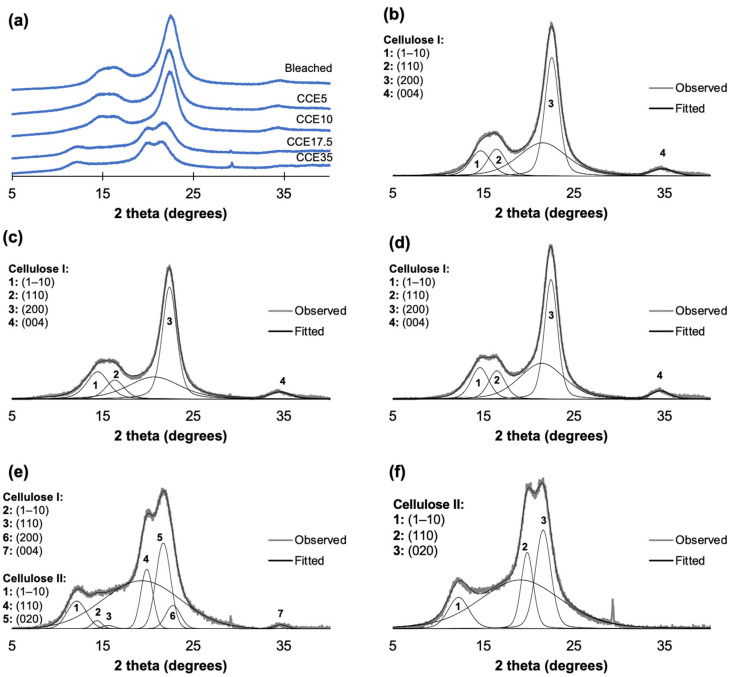
Pine XRD diffractograms. (**a**) XRD diffractograms of pine pulp after alkaline treatment, (**b**) curve-fitting of bleached kraft pulp, (**c**) curve-fitting of CCE5, (**d**) curve-fitting of CCE10, (**e**) curve-fitting of CCE17.5, and (**f**) curve-fitting of CCE35 pine sample.

The XRD patterns of eucalyptus and pine kraft pulps are shown in Figure 3 and Figure 4. From the diffraction pattern of cellulose I, the 14.8°, 16.5°, and 22.3° 2θ reflections are assigned to the (1–10), (110), and (200) crystallographic planes, respectively. The 18.5° 2θ reflection is assigned to the amorphous phase, and the 34.5° 2θ reflection is assigned to the (004) plane [45,46]. From the diffraction pattern of cellulose II, the 12°, 20°, and 21.6° 2θ reflections are assigned to the Miller indices of (1–10), (110) and (020), respectively [45]. Eucalyptus, bleached, and CCE5 pulps showed reflections associated with the crystallographic planes of cellulose I (Figure 3b,c) [45]. CCE10 also showed the typical XRD pattern of cellulose I, but small reflections start to emerge around 12° and 20° 2θ (Figure 3d), indicating the presence of cellulose II [45]. After CCE17.5 treatment, the diffraction pattern indicated a major fraction of cellulose II, while cellulose I reflections are still present (Figure 3e). After CCE35 treatment, cellulose I was completely converted to cellulose II (Figure 3f). Regarding pine samples, bleached, CCE5 and CCE10 samples showed reflections associated with cellulose I (Figure 4b–d). CCE17.5 pine sample showed reflections associated with cellulose II and small reflections associated with cellulose I (Figure 4e), while CCE35 was completely converted to cellulose II (Figure 4f).

**Figure 5:**
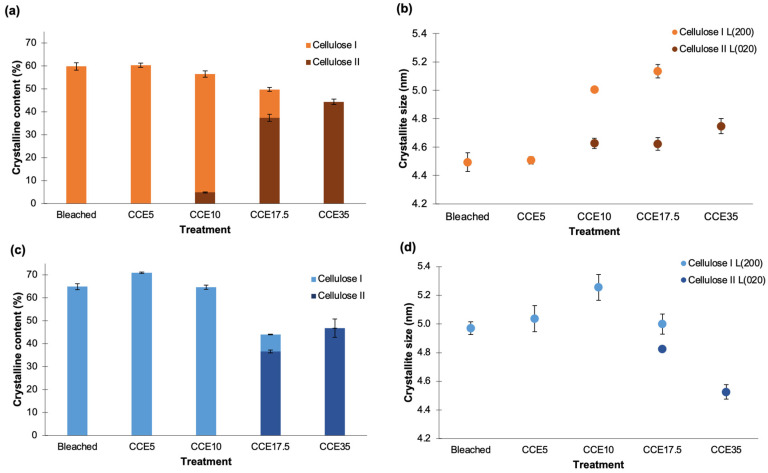
Crystalline structure of cellulose. (**a**,**c**) Crystallinity and (**b**,**d**) crystallite size for eucalyptus and pine pulps, respectively. Different colors in bars indicated proportions of cellulose I and II in samples.

The crystallinity index (CrI) was determined using the deconvolution method [39], and the profiles were resolved into four crystalline contributions for cellulose I, three crystalline contributions for cellulose II, and into a broad band as the amorphous contribution (Figure 3 and Figure 4) [24]. Figure 5 shows the CrI and lateral crystallite size (*L*) obtained for eucalyptus (Figure 5a,b) and pine (Figure 5c,d). Eucalyptus CrI ranged from 60 to 44%, decreasing as alkali treatment proceeded. The 52% of the total crystalline proportion of eucalyptus CCE10 sample corresponded to cellulose I and 5% to cellulose II, while CCE17.5 sample resulted in 12% of cellulose I and 37% of cellulose II. The CrI of pine samples ranged from 71 to 44%, whose values also decreased as the alkali treatments proceeded. In the case of pine CCE17.5 sample, the 37% of the total crystalline fraction corresponded to cellulose II (same as eucalyptus CCE17.5 sample) and 7% to cellulose I. Regarding crystallite sizes, *L*_(200)_ of cellulose I ranged from 4.5 to 5.1 nm in eucalyptus pulps and from 5.0 to 5.3 nm in pine, while *L*_(020)_ of cellulose II spanned 4.6–4.7 nm in eucalyptus, and 4.5–4.8 nm in pine. The increase of *L*_(200)_ after CCE10 and CCE17.5 can be promoted by a co-crystallization of the cellulose within a bigger crystallite by the joining or coalescence of two or more crystals. Since the alkaline treatments mostly remove hemicelluloses and low molecular weight disordered cellulose chains [47], the co-crystallization phenomenon may explain the increasing lateral crystallite size in the cellulose I polymorph [48]. On the other hand, the pine sample, which has a larger size of cellulose I crystallites than eucalyptus, was converted to cellulose II at higher alkali concentration (CCE17.5) than eucalyptus (CCE10), probably because a larger number of lattice units need to be swollen for NaOH penetration.

### 3.4. Intrinsic Viscosity

**Figure 6:**
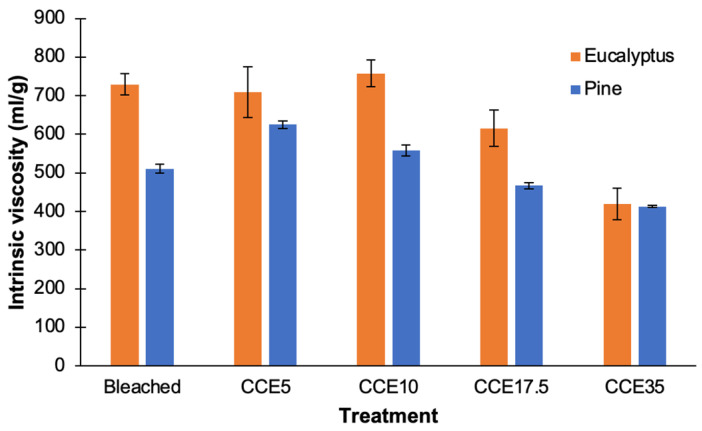
Intrinsic viscosity of bleached and CCE-treated kraft pulps from eucalyptus and pine.

Bleached eucalyptus presented intrinsic viscosity of ~730 mL/g. After CCE5 and CCE10 treatment, negligible variation on viscosity was observed (Figure 6). Similar observations were previously described in bleached kraft pulps from different eucalyptus species at the same CCE conditions [14]. After CCE17.5 and CCE35 treatments, the intrinsic viscosity decreases to 615 and 420 mL/g, respectively. It means that, over 17.5% NaOH (*w/v*) concentration, alkali treatment was able to cleave glycosidic linkages of cellulose, reducing its polymerization degree [49].

Bleached pine pulp presented an intrinsic viscosity of ~510 mL/g and after CCE5 it was increased to 625 mL/g (Figure 6). This change agrees with the results presented in Figure 1b, assuming that broken fibers with shorter cellulose chains are dissolved by the CCE5 treatment and the proportion of longer cellulose chains increased, as does the intrinsic viscosity of pulps. After CCE17.5 and CCE35 treatments, the intrinsic viscosity decreases to 470 and 410 mL/g, respectively. As also observed for eucalyptus pulps, treatment with NaOH concentrations over 17.5% NaOH (*w/v*) can significantly reduce the polymerization degree of cellulose.

### 3.5. Carboxyl Content

Negative charges in fibers are generated during alkaline pulping of wood. It is known that hemicelluloses from eucalyptus and pine behave differently during kraft pulping. Xylans are retained in the pulp since they are less degraded and more stable than glucomannans. This is due to the stabilizing effect of the methylglucuronic acid (MeGlcA) side-groups that stop peeling reactions in the reducing ends of the xylans chains. MeGlcA are also converted to hexenuronic acids (HexA) in alkaline medium [50] which are only partially removed during bleaching. Kraft pulping also results in the formation of polysaccharide carboxyl groups, generated from the peeling reaction of cellulose, which is stopped by the formation of metasaccharinic acid or other alkali-stable carboxyl groups. Oxidative bleaching agents such as chlorine derivatives can also generate carboxyl groups in cellulosic fibers [50,51,52,53]. Hence, bleached eucalyptus kraft pulps contained a higher initial amount of negative charges than pine pulps, as mannans do not have a significant amount of acid side-groups. In bleached pine pulps, carboxyl content may be mainly attributed to oxidized polysaccharides [54,55].

**Figure 7:**
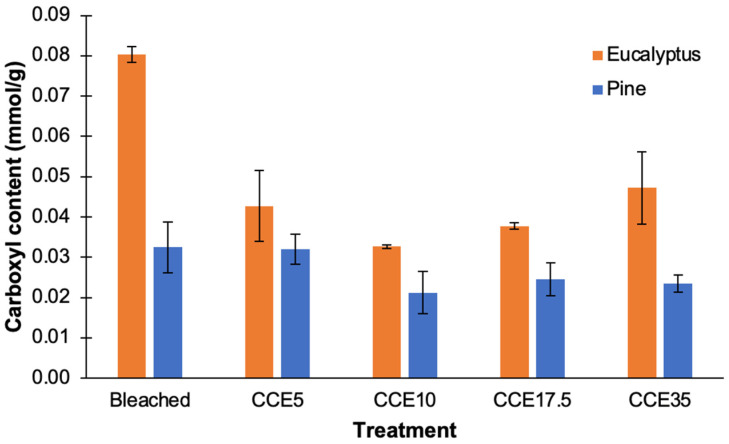
Carboxyl content of bleached and CCE-treated kraft pulps from eucalyptus and pine.

Bleached eucalyptus pulp presented carboxyl levels of 0.080 mmol/g, whose content was diminished to 0.043 and 0.033 mmol/g after CCE5 and CCE10 treatments, respectively. CCE17.5 and CCE35 presented a carboxyl content of 0.038 and 0.047 mmol/g, respectively (Figure 7). On the other hand, the shorter cellulose chains of CCE17.5 and CCE35 samples also contributed to increase the access to the internal structure of fibers allowing the diffusion of ions involved in the conductimetric titration [38]. The amount of carboxyl groups in pine (from 0.032 to 0.021 mmol/g) was significantly lower than eucalyptus. With the CCE extraction, the carboxyl content in eucalyptus pulps is decreased, mainly due the removal of xylans. In pine there is only a slight decrease in the carboxylic groups. We can hypothesize that the low amount of carboxyl groups found in eucalyptus and pine pulps after CCE extraction are mainly from some residual oxidized anhydroglucose units in the cellulose chains.

### 3.6. Influence of CCE Treatment on Pulp Features

To define the most relevant influence of CCE treatments on fibers, Pearson correlation indexes were established among the investigated variables. Since fiber morphology is different between eucalyptus and pine, correlation indexes were determined separately for each species. Table 2 and Table 3 shows the correlation indexes among variables from eucalyptus and pine samples, respectively. Significant correlations were observed between CrI vs. intrinsic viscosity and fiber width; fiber length vs. SSA; kink index vs. carboxyl content; and xylans content vs. SSA, fiber length, fiber width and kink index in both species.

### 3.7. Enzymatic Multicomponent Saccharification of Pulps

**Figure 8:**
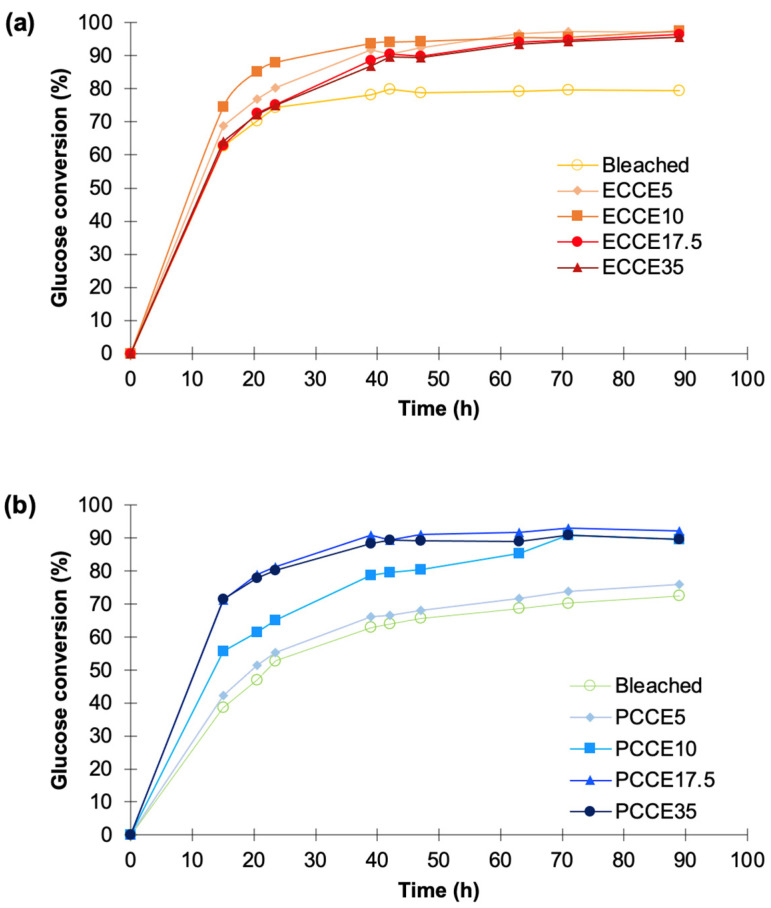
Enzymatic multicomponent (Cellic CTec3) hydrolysis of (**a**) eucalyptus and (**b**) pine fibers after CCE treatments.

After 15 h of treatment, the glucans to glucose conversion ranged from 62% to 74% in eucalyptus, being lowest in bleached eucalyptus pulp and highest in the CCE10 pulp (Figure 8a). After 40 h, the bleached sample reached to the maximum conversion yield (~79.5%) while CCE5, CCE10, CCE17.5, and CCE35 reached to 87–93%. For pine pulps, the glucose conversion ranged 38–71% after 15 h, being lowest in bleached pine pulp and highest in CCE17.5 and CCE35 (Figure 8b). After 50 h, the CCE17.5 and CCE35 reached to the maximum conversion yield (~90%), while the bleached pine pulp registered the higher conversion after 90 h (72%), being followed by CCE5 sample (76%) and CCE10 (90%).

Monomeric sugars such as xylose and mannose have been claimed to decrease the hydrolytic potential of cellulases as they reduce the productive binding of the cellulase domain on cellulose surfaces [17,56]. This might be one of the reasons that explain the lower conversion values obtained for kraft pulps with hemicelluloses in their composition (Table 1). It is noteworthy that the Cellic CTec3 cocktail also has high endoxylanase activity, which can promote xylan degradation in eucalyptus pulps, contributing to increased cellulases access to fibers and higher glucose yields from CCE-treated pulps. On the other hand, crystalline cellulose II is known to have a higher susceptibility to enzymatic hydrolysis than the cellulose I polymorph [57]. However, only pine samples with cellulose II (CCE17.5 and CCE35) showed higher susceptibility to saccharification than those with cellulose I.

It is known that cellulose crystals are composed of several planes with different hydrophobicities and that the adsorption of cellulase-binding domain onto cellulose occurs through hydrophobic interactions [2,57,58,59,60,61]. The hydrophobic crystalline face of cellulose I and cellulose II are the (200) and (110) planes, respectively. Smaller d-spaces are typically observed in the hydrophobic plane of cellulose I, which indicates stronger hydrophobic interactions in the stacked chains of cellulose I [57,61]. This stronger interaction contributes to a lower efficiency of the hydrolytic enzyme, while weaker hydrophobic interactions of cellulose II contribute to the better efficiency of enzymatic hydrolysis [57,61]. To evaluate this approach, lateral crystallite sizes and d-spacing of hydrophobic planes of cellulose were determined (Table 4). It can be observed that cellulose I presented smaller d-spacing of the hydrophobic plane than cellulose II in both species, and that the lateral crystallite size of the hydrophobic planes of cellulose II are higher than the ones of cellulose I. It means that the lattice spacing and crystallite size of cellulose II provide more space for enzyme access and binding [61]. These results explain the observation of higher glucose conversion of cellulose II crystalline fractions from pine samples (CCE17.5 and CCE35) than the fractions of cellulose I from pine. However, it does not explain the conversion results of eucalyptus. 

**Figure 9:**
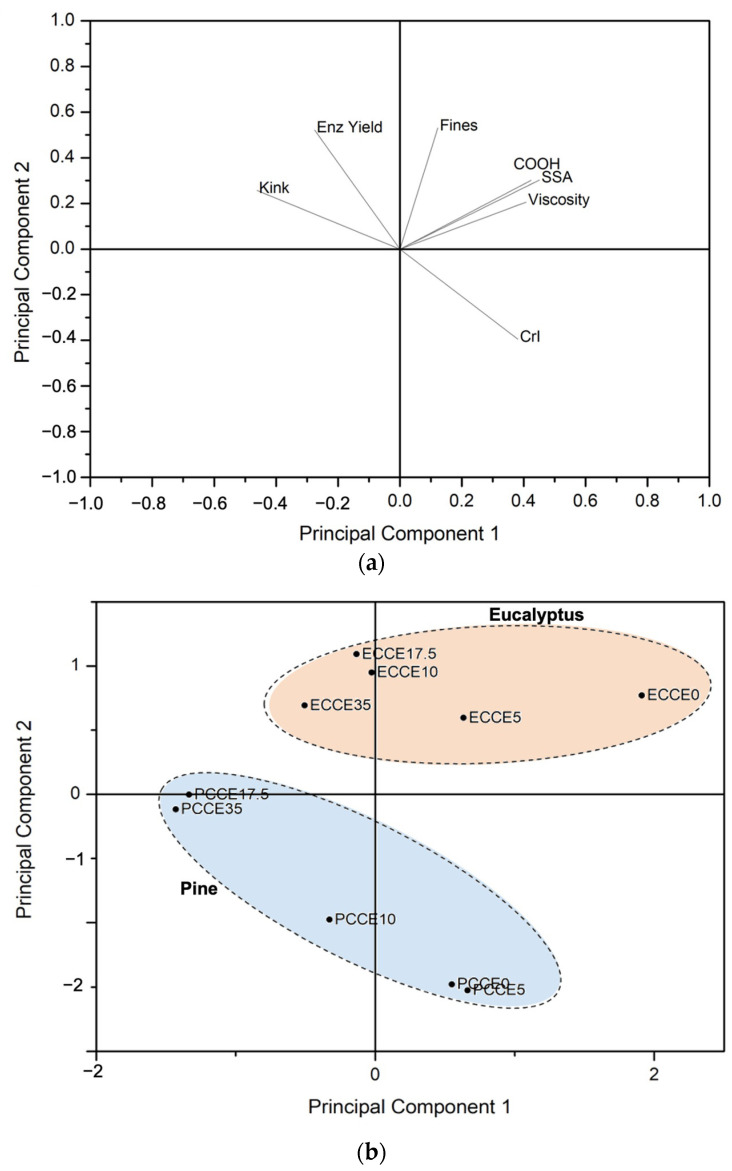
Principal component analysis. (**a**) Loading graph showing the contribution of variables to the PC1 and PC2. (**b**) Score graph showing the observations on the coordinates of the PC1 and PC2.

To integrate the variables evaluated and determine those most significant for the results obtained, a Principal Component Analysis (PCA) was performed (Figure 9). The variables included were kink index (kink), fines content (fines), carboxyl content (COOH), specific surface area (SSA), intrinsic viscosity (viscosity), crystallinity index (CrI), and the glucose yield after 40 h of saccharification (Enz Yield). The PCA was constructed using two main components, which explained 78.94% of the total variation in the data. The loading plot (Figure 9a) shows that the first component (PC1) explains the variation in kink index, SSA, carboxyl content, and intrinsic viscosity, while the second component (PC2) explains the variation of fines content, CrI and glucose conversion. Figure 9b shows the grouping of eucalyptus and pine samples in relation to the two main components. Most of the eucalyptus samples showed scores around PC1, while in PC2, they are relatively constant. Regarding pine samples, their scores clearly show that as CCE treatments proceeded, CrI values decreased, and enzymatic hydrolysis yield and kink index increased. The correlation indexes determined for pine samples were significant for CrI (r = −0.95, *p*-value = 0.003) and kink index (r = −0.90, *p*-value = 0.015) vs. enzymatic hydrolysis yield. However, these correlations could not be established in eucalyptus samples.

### 3.8. Endoglucanase (EG) Treatment of Pulps for Viscosity Control

The susceptibility of CCE-treated pulps to EG treatment was investigated for intrinsic viscosity control aimed to dissolving grade pulp production. EG have been previously investigated as an environmentally friendly step to upgrade paper-grade pulp to dissolving grade cellulose [19,20,62,63]. Intrinsic viscosity, an indirect measurement of the polymerization degree, is one the key quality parameters of dissolving-grade pulps for cellulose derivatives production [15], e.g., for viscose, values are around 400–600 mL/g [14,64], for cellulose acetate values are ~650–800 mL/g [15,64], and for cellulose ethers ~470–600 mL/g [64,65].

**Figure 10:**
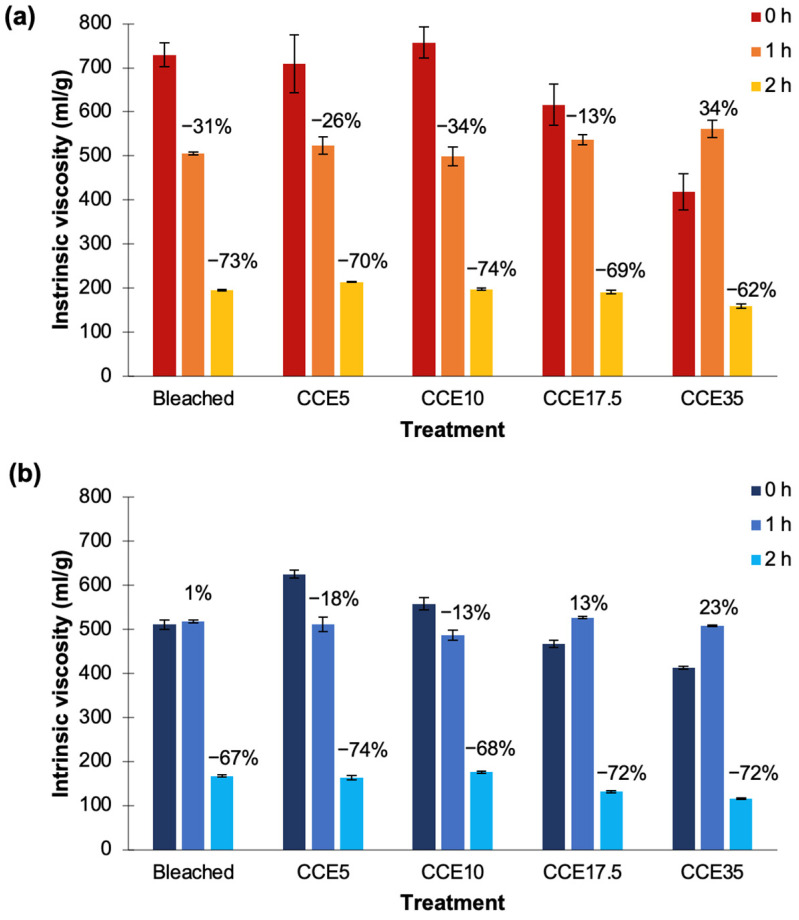
Variation of the intrinsic viscosity after EG treatments in (**a**) eucalyptus and (**b**) pine pulps. Dark blue: pulps without EG treatment. Light blue: EG treatment with enzyme load of 5 ECU/g pulp for 1 h. Gray: EG treatment with enzyme load of 10 ECU/g pulp for 2 h.

Two EG conditions were tested in order to determine the intrinsic viscosity behavior of pine and eucalyptus when treatments with different severity are used; enzyme load of 5 ECU/g pulp for 1 h and enzyme load of 10 ECU/g pulp for 2 h. In bleached, CCE5, and CCE10 eucalyptus samples, the intrinsic viscosity values were reduced by ~30% after 1 h of EG treatment. In CCE17.5, the values were reduced by only 13%, while in CCE35 the intrinsic viscosity increased 140 units (Figure 10a). During this treatment, the highest value was obtained for CCE35 (560 mL/g) and the lowest was CCE10 (500 mL/g) among eucalyptus samples. After 2 h of EG treatment, intrinsic viscosities were reduced by ~70%, ranging from 160 to 215 mL/g. The most susceptible Eucalyptus sample to both EG treatments was CCE10-treated sample, while the most resistant was CCE17.5-treated sample after 1 h, and CCE35 after 2 h (Figure 10a).

Regarding pine samples, the bleached sample after 1 h of EG treatment showed no variation in comparison to the untreated sample (Figure 10b). CCE5 and CCE10 pine samples reduced viscosity in 115 and 54 units, respectively, while CCE17.5 and CCE35 increased in 56 and 100 units, respectively. During this treatment, the highest viscosity value was obtained for CCE17.5 (525 mL/g), and the lowest was CCE10 (490 mL/g) among pine samples. After 2 h of EG treatment, the intrinsic viscosities were reduced from 67 to 74%, ranging from 115 to 176 mL/g. The most susceptible pine sample to both EG treatments was CCE5-treated sample, while the most resistant was the bleached pine sample (Figure 10b). Previous studies have shown that CCE treatments can aid the viscosity reduction mediated by EG treatment [13,63,66,67,68].

When CCE is used to upgrade paper-grade pulp to dissolving-grade, precautions must be taken regarding the NaOH concentration. The partial conversion of cellulose I to cellulose II have been reported detrimental to pulp reactivity [12,13], since when alkalized fibers are dried, hornification occurs and cellulose becomes less accessible and less reactive due to the collapse of interfibrillar spaces, resulting in a dried pulp with decreased surface area and pore volume [13,62]. On the contrary, when wet mercerized fibers (cellulose II hydrate) are used, the reactivity and accessibility are significantly superior [57]. In this work, bleached and CCE samples used were never-dried pulps. However, no evident influence of the hydrated cellulose II crystal system was observed after EG treatment during 1 h. After EG treatment during 2 h, the samples with total cellulose II conversion (CCE35) were the ones with lowest intrinsic viscosity values achieved in eucalyptus (160 mL/g) and pine (115 mL/g).

Regarding viscosity of eucalyptus, bleached and CCE5 samples are suitable for cellulose acetate products, while CCE17.5 and CCE35 after EG (1h) are suitable for cellulose ethers. CCE35 is adequate for the lyocell process. Bleached, CCE5, CCE10, and CCE17.5 samples subjected to EG treatment by 1 h are suitable for viscose products. Regarding pine samples, most samples are suitable for viscose and lyocell products, with the exception of CCE5 that could be used for nitrate or ether products. Regarding the content of hemicelluloses, eucalyptus CCE10, CCE17.5, and CCE35 samples, and the pine CCE17.4 and CCE35 samples show adequate values for high purity dissolving pulp (hemicelluloses <1.5%) [15,64].

**Figure 11:**
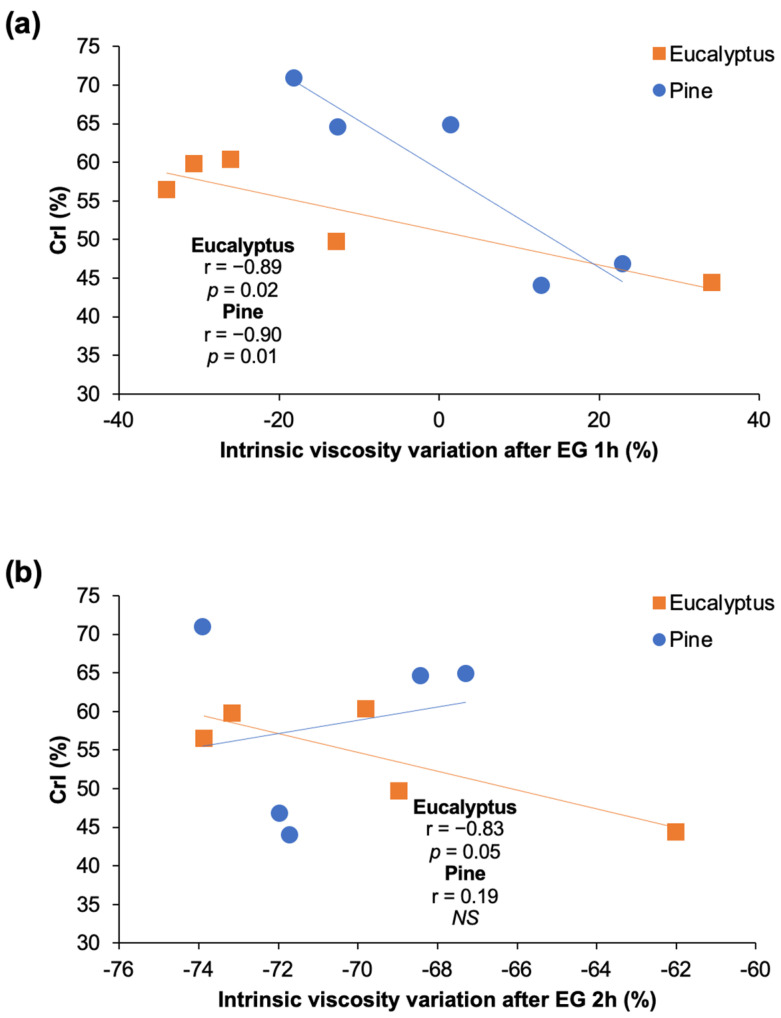
Regression lines and Pearson correlation indexes (r) of crystallinity degree vs. variation of the intrinsic viscosity after EG treatment of eucalyptus and pine pulps for (**a**) 1 h and (**b**) 2 h. Correlations were considered significant at *p* value < 0.05. NS: not significant.

Viscosity variations in pulps is dependent on the EG accessibility to cellulose chains. First, EG act on less ordered cellulose regions and a fast reaction takes place on the most accessible amorphous portions, afterwhile, a slow degradation of the crystalline portion may occur depending on the porosity and accessibility [67,68,69]. Significant correlations (at *p* < 0.05) were established between the CrI of eucalyptus and pine samples vs. the variation of the intrinsic viscosity promoted by the EG treatment during 1 h (Figure 11a). Hence, it is stated that a higher CrI decreases the depolymerization of cellulose by EG. After 2 h of EG treatment, significant correlations among CrI and viscosity changes were established only for eucalyptus (Figure 11b). On the other hand, it has been stated that the morphology of fiber surface and ultrastructure (such as pore volume, micropore, and macropore amount) would facilitate the enzyme adsorption and penetration into the cell wall, thus the viscosity decrease mediated by EG [68,70]. In this work, pore parameters were not measured, however, similar morphological features such as SSA, kink index, or fines content did not show a significant influence on EG hydrolysis. Overall, it was observed that eucalyptus samples showed higher susceptibility to the EG treatment than pine samples, that was mainly related to the low crystallinity of the pulps.

## 4. Conclusions

Bleached kraft pulps from eucalyptus and pine were subjected to CCE to determine the influence of alkali concentration on fiber biometry, hemicelluloses removal, and crystalline structure of cellulose. Samples were further submitted to enzymatic treatment for saccharification (glucose production) or viscosity control (dissolving grade pulp production).

(1) CCE treatment was able to generate modifications in chemical composition and crystallinity of eucalyptus and pine bleached kraft pulps. NaOH concentrations above 17.5% cause a depolymerization of the cellulose chains, a decrease in fiber length and in CrI. Furthermore, eucalyptus started the conversion of cellulose I to cellulose II at NaOH 10%, while in pine it occurs at 17.5% NaOH.
(2) Enzymatic saccharification yields higher than 90% where obtained with CCE-treated eucalyptus pulps while in bleached pulp it was 80%. In pine, only CCE-treated pulps with higher proportion of cellulose II showed saccharification yields higher than 90%.
(3) CCE combined with EG treatment is an efficient method to control the intrinsic viscosity of pulps. The eucalyptus samples showed higher susceptibility to the EG treatment than the pine samples, which was mainly related to the low crystallinity of the pulps.
(4) Overall, eucalyptus pulps are more accessible and reactive than pine pulps. The objective of increasing the reactivity, converting cellulose I to cellulose II hydrate, proved to be relevant only for the saccharification of pine pulps.

## Figures and Tables

**Table 1:** Chemical composition and fiber biometry of eucalyptus and pine pulps. Mean ± standard deviation of each variable is presented.

Sample	Treatment	Glucans (%)	Xylans ^1^ or Mannans ^2^ (%)	Fiber Length (mm)	Fiber Width (um)	Fines Content (%)	Mean Kink Index
**Eucalyptus ^1^**	**Bleached**	90.2 ± 1	9.8 ± 0.2	0.69 ± 0.01	18.1 ± 0.1	4.4 ± 0.1	3 ± 0
	**CCE5**	93.6 ± 1	6.5 ± 0.1	0.647 ± 0.004	18.1 ± 0.1	4.5 ± 0.2	3.8 ± 0.3
	**CCE10**	>99	<1	0.575 ± 0.001	18 ± 0	5.8 ± 0.3	3.79 ± 0.01
	**CCE17.5**	>99	<1	0.536 ± 0.004	17 ± 0	7 ± 1	4 ± 0.03
	**CCE35**	>99	<1	0.59 ± 0.08	17.3 ± 0.4	5 ± 1	3.83 ± 0.03
**Pine ^2^**	**Bleached**	92 ± 1	7.5 ± 0.4	2.224 ± 0.004	32.5 ± 0.2	3.1 ± 0.1	3 ± 0.1
	**CCE5**	93.2 ± 0.2	6.5 ± 0.2	2.19 ± 0.02	32.5 ± 0.1	2.3 ± 0.1	3.3 ± 0.1
	**CCE10**	94 ± 3	5.5 ± 1.1	2.031 ± 0.001	33.1 ± 0.1	2.5 ± 0.1	3.99 ± 0.04
	**CCE17.5**	>99	<1	1.79 ± 0.03	31.6 ± 0.4	3 ± 1	4.1 ± 0.4
	**CCE35**	>99	<1	1.74 ± 0.02	31.5 ± 0.5	2.4 ± 0.4	4.2 ± 0.1

^1^ and ^2^ represent the main residual hemicellulose in eucalypts or pine pulps, respectively.

**Table 2:** Pearson correlation index between evaluated variables of the eucalyptus samples. *n* = 5.

	Carboxyl Content	Intrinsic Viscosity	CrI	SSA	FL	FW	Fines	Kink Index	Xylans Content	Glucans Content
**Carboxyl content**	1	0.11	0.32	0.85 *	0.80	0.47	−0.65	−0.93 *	0.83 *	−0.83 *
**Intrinsic viscosity**		1	0.91 *	0.37	0.38	0.79	−0.15	−0.38	0.48	−0.48
**CrI**			1	0.63	0.69	0.96 *	−0.52	−0.55	0.74	−0.74
**SSA**				1	0.88 *	0.70	−0.72	−0.81	0.97 *	−0.97 *
**FL**					1	0.84 *	−0.94 *	−0.87 *	0.95 *	−0.95 *
**FW**						1	−0.73	−0.69	0.83 *	−0.83 *
**Fines**							1	0.69	−0.81	0.80
**Kink index**								1	−0.85 *	0.86
**Xylans content**									1	0.99 *
**Glucans content**										1

* Indicates significant correlation at *p* < 0.05.

**Table 3:** Pearson correlation index between evaluated variables of the pine samples. *n* = 5.

	Carboxyl Content	Intrinsic Viscosity	CrI	SSA	FL	FW	Fines	Kink Index	Xylans Content	Glucans Content
**Carboxyl content**	1	0.45	0.56	0.52	0.75	0.13	0.37	−0.93 *	0.65	−0.63
**Intrinsic viscosity**		1	0.89 *	0.78	0.80	0.77	−0.25	−0.59	0.77	−0.76
**CrI**			1	0.98 *	0.94 *	0.85 *	0.01	−0.75	0.95 *	−0.95 *
**SSA**				1	0.91 *	0.84 *	0.08	−0.73	0.95 *	−0.95 *
**FL**					1	0.76	0.31	−0.92 *	0.99 *	−0.98 *
**FW**						1	0.07	−0.45	0.83	−0.84 *
**Fines**							1	−0.48	0.32	−0.33
**Kink index**								1	−0.86 *	0.85 *
**Xylans content**									1	0.98 *
**Glucans content**										1

* Indicates significant correlation at *p* < 0.05.

**Table 4:** Lateral crystallite size and d-spacing of hydrophobic planes of cellulose I (200) and cellulose II (110) in eucalyptus and pine samples. Standard deviation lower than 2%.

Specie	Treatment	Lateral Crystallite Size (nm)		D-Spacing (Å)	
		CI ^1^ (200)	CII ^2^ (110)	CI ^1^ (200)	CII ^2^ (110)
**Eucalyptus**	**Bleached**	4.49	-	3.95	-
	**CCE5**	4.51	-	3.94	-
	**CCE10**	5.01	6.79	3.94	4.40
	**CCE17.5**	5.13	5.77	3.93	4.44
	**CCE35**	-	6.29	-	4.42
**Pine**	**Bleached**	4.97	-	3.95	-
	**CCE5**	5.04	-	3.98	-
	**CCE10**	5.26	-	3.96	-
	**CCE17.5**	5.00	5.82	3.91	4.47
	**CCE35**	-	5.70	-	4.57

## Data Availability

The data presented in this study are available on request from the corresponding author.
